# Migrant GPs and patients: a cross-sectional study of practice characteristics, patient experiences and migration concordance

**DOI:** 10.1080/02813432.2022.2069719

**Published:** 2022-05-14

**Authors:** Peter P. Groenewegen, Peter Spreeuwenberg, A. Niroshan Siriwardena, Coral Sirdifield, Sara Willems

**Affiliations:** a Nivel, The Netherlands Institute for Health Services Research, Utrecht, The Netherlands; b Department of Sociology and Department of Human Geography, Utrecht University, Utrecht, The Netherlands; c Community and Health Research Unit, School of Health and Social Care, University of Lincoln, Lincoln, UK; d Department of Public Health and Primary Care, Ghent University, Gent, Belgium

**Keywords:** Concordance, discrimination, health service research, family practice, patient experiences, immigrant GPs, migrant patients

## Abstract

**Objective:**

To investigate practice type and location of native and immigrant general practitioners (GPs); effects of migration status concordance between GPs and patients on experiences of patients in key areas of primary care quality and discrimination.

**Design and setting:**

Secondary analysis of GP and patient survey data from QUALICOPC (Quality and Costs of Primary Care), a cross-sectional study of GPs and their patients in 34 countries, performed between 2011 and 2013.

**Main outcome measures:**

We explored practice type and location of native and immigrant GPs and the experiences of native patients and patients with a migration background of communication, continuity, comprehensiveness, accessibility, and discrimination, using multilevel analysis. Concordance was modelled as a cross-level interaction between migration status of GPs and patients.

**Results:**

Percentages of immigrant GPs varied widely. In Europe, this was highest in England and Luxemburg (40% of GPs born abroad) and lowest in Bulgaria and Romania (1%). The practice population of immigrant GPs more often included an above average proportion of people from ethnic minorities. There were no differences in main effects of patient experiences following a visit to an immigrant or native GP, in four core areas of primary care or in discrimination. However, people from first-generation migrant background more often experienced discrimination, in particular when visiting a native GP.

**Conclusion:**

Patient experiences did not vary with GPs’ migration status. Although experience of discrimination was uncommon, first-generation migrant patients experienced more discrimination. Primary care should provide non-discriminatory care, through GP awareness of unconscious bias and training to address this.

**Key messages**
- There were large differences in percentage of migrant GPs between countries.
- Migrant GPs’ practices had an above average proportion of people from ethnic minorities.
- In general, patients’ experienced discrimination from GPs and practice staff was low, but first-generation migrant patients more often experienced discrimination.
- First-generation migrant patients more often experienced discrimination when they visited a native GP.

## Introduction

Global migration has led to increases in doctors and patients with migration backgrounds [1]. They may experience difficulties in their host country, including communication problems, cultural differences, or discrimination. This is the case among both the general population (for example in their experience of health care [2] or the labour market [3]) and healthcare professionals with a migration background [4].

Through explicit policies, personal decisions or other processes, immigrant general practitioners (GPs) often practise in underserved areas. They are more likely to work in urban areas and serve communities with greater migration backgrounds [5,6]. Some countries have policies to attract foreign doctors to underserved or rural areas (for example in Germany [7] and the UK [8]), but the urban-rural distribution of immigrant GPs is unclear [6]. Our first research question is therefore: *Where do immigrant GPs practise (practice location and type of practice)? Do they have more patients with a migration background?*

Physician migration may affect macro level health system performance positively (e.g. working in underserved areas) or negatively (e.g. specific skill deficits) [7]. At the micro level, language and cultural differences or similarities may affect care quality. Patient experience of primary care quality varies widely between GPs and countries, in access, continuity, coordination, and doctor-patient communication [9]. Data from the QUALICOPC (Quality and Costs of Primary Care) study showed patients with migration backgrounds, particularly first-generation, experienced poorer quality care [10]. Discrimination was experienced by primary care patients in many countries to varying degrees, again, particularly by first generation migrants [11]. Patient experiences are based on survey questions that ask for concrete experiences in the consultation with a GP, for example ‘The doctor or staff acted negatively to you’, with the answering options yes or no.

The situation may differ when both GP and patient are born abroad. GPs and patients sharing sociodemographic characteristics is termed concordance. Gender concordance, for example, affects doctor-patient communication, patient-centredness and treatment effectiveness, according to a brief overview of the literature in an empirical paper on gender concordance and antibiotic prescribing [12]. Ethnic and migration background concordance have shown variable effects. A systematic review of racial concordance (the term used in the title of the paper) showed effects on several aspects of doctor-patient communication, such as patient satisfaction and participatory decision-making, but not communication quality [13]. No effect of racial/ethnic concordance (term used in the title of the papers) was found on uptake of primary care prevention [14] or satisfaction [15]. Positive effects of immigrant status concordance (term used in the title) may be due to a different approach to cultural differences among immigrant GPs [16]. Hence, concordance effects may occur, when both patient and GP are from the same country or culture but also when both have a migration background. We investigate the latter in this study. Our second question is therefore: *How do patients experience the care they receive from immigrant GPs? Does migration status (dis)concordance of GPs and patients affect patient experiences?*

## Material and methods

### QUALICOPC study

We report on a secondary analysis of cross-sectional data from the QUALICOPC study. Data were collected between 2011 and 2013 from approximately 7,200 GPs and 63,500 patients in 31 European and three non-European countries. In each country, a sample of around 220 GPs completed a questionnaire, except for four smaller countries where this was around 75 GPs. As far as possible, random samples were drawn. Per practice, only one GP was asked to participate [17]. The GP questionnaire asked about practice organisation and context.

Trained fieldworkers invited consecutive patients to complete a questionnaire about their experiences as they left the consultation, until nine questionnaires were collected. Questions were derived from other validated questionnaires. Details of study design and questionnaire development can be found elsewhere [18,19].

### GP and practice characteristics

The main GP variable was whether they were born abroad. Information on practice location was derived from a subjective assessment of urbanity. Practice composition was measured as a subjective assessment of the proportion of elderly people, people from ethnic minorities, and deprived people (above average, average, below average). Finally, we considered whether the GPs worked in a shared (group) or single-handed practice.

### Patient experiences and characteristics

Patient experiences were assessed in four areas of primary care quality: communication, continuity, comprehensiveness, and accessibility, using scales [9]. Additionally, the questionnaire asked about experienced discrimination in the past 12 months through three items, combined into a scale: The doctor or staff acted negatively to you; Other patients were treated better than you; The doctor or staff showed disrespect because of your ethnic background. These items were taken from the Commonwealth Survey [20]. Answering options were yes, no and don’t know.

Patients were also asked where they and their mother were born. When patient and mother are born in the country of residence they were considered ‘native’. When patients are born elsewhere, they were considered first generation migrants and when the mother was born elsewhere and the patient born in the country of residence, s/he was considered a second-generation migrant [10].

We used the following confounders at patient level: self-reported health, whether or not patients had a chronic condition, sex, age, level of education and household income, because of their known association with patient experiences.

### Data analysis

We performed multilevel analysis to take the nested character of the data into account, with patients nested in GPs and GPs in turn nested in countries [21]. Missing values were excluded listwise. The analysis corresponds to the two research questions.

For research question one, we estimated the percentage of immigrant GPs in separate two-level (GPs within countries) logistic multilevel models for practice location, practice composition and practice type, controlled for GP age and sex.

For research question two, patient experiences were the dependent variables in three-level (patients, GPs, countries) linear multilevel analyses. Patient experience scales for the four areas of primary care have been described elsewhere [9]. The experience of discrimination scale was built in a four-level (items, patients, GPs, countries) multilevel model. Reliability was calculated at patient, GP and country levels [22].

We first estimated a null model (Model 0; only constant and variances). Model 1 includes whether GPs were born abroad. Model 2 additionally includes the GPs’ age, sex, and practice location variables. Model 3 additionally includes the patient variables. Finally, in Model 4, we added the cross-level interaction of GP and patient migration status.

The experience of discrimination items contained many ‘don’t knows’ and a very skewed distribution of responses. We therefore performed two sensitivity analyses; the first included only GPs with at least one patient with a migration background among the responders; the second used the single item with the highest percentage of patients reporting discrimination and the lowest number of ‘don’t knows’ (‘The doctor or staff acted negatively to you’) as the dependent variable.

Data were analysed using MLwiN, version 2.30.

## Results

### Practice type and location

**Figure 1.**
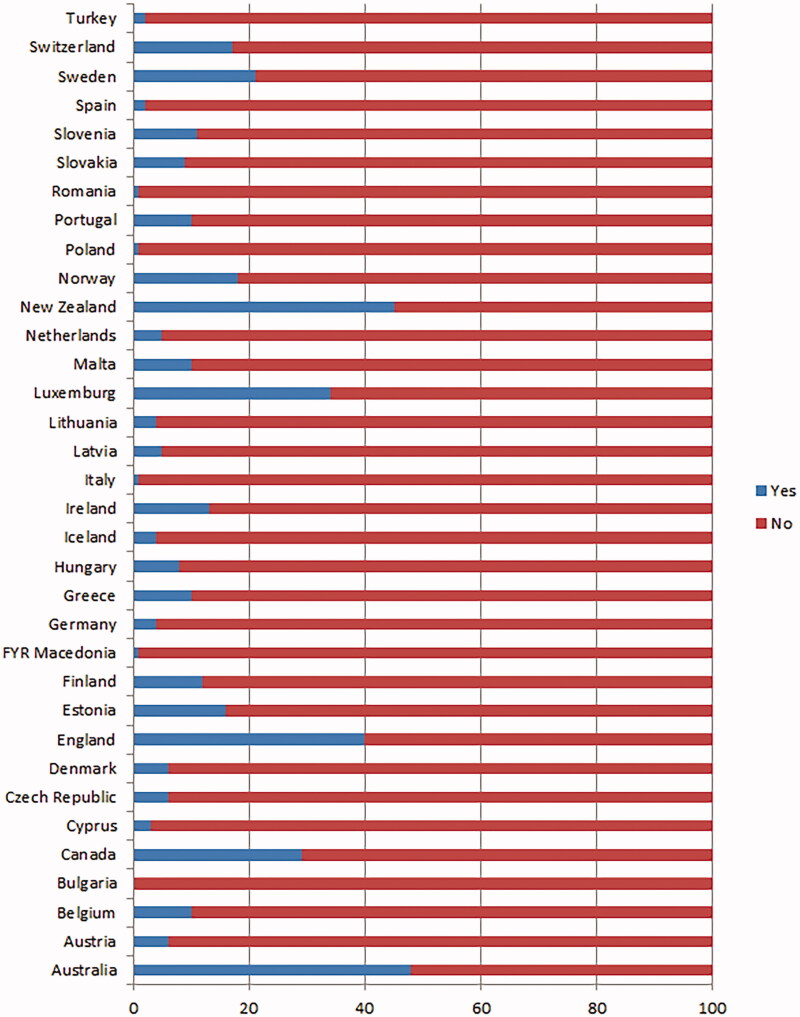
Percentage of GPs born in another country (Yes) than where they practise (N_countries_ =34; N_GPs_ =7,182).

Overall, the percentage of immigrant GPs in the countries studied averaged 12% with large differences between countries (Figure 1), varying from zero in Bulgaria to 48% in Australia and 40% in England.

The practice population of immigrant GPs more often had an above average proportion of minority ethnic people (*p*=.04) compared with native doctors (Table 1). There were no significant differences in other aspects of practice location or type.

**Table 1.** Practice location and practice type of immigrant GPs (estimates based on separate 2 level multilevel logistic regression models for each independent variable, controlling for age and sex of GPs).

Variable	Percentage immigrant GPs	Significant difference from overall average
Urbanisation		
(N_countrie_*_s_* =34; N_GP_*_s_* =7,182)		
- big (inner) city	8.7%	ns
- suburbs	7.4%	ns
- (small) towns	8.5%	ns
- mixed urban-rural	10.7%	ns
- rural	9.4%	ns
Elderly in practice population		
(N_countrie_*_s_* =34; N_GP_*_s_* =7,006)		
- below average	8.8%	ns
- average	8.1%	ns
- above average	7.8%	ns
Ethnic minority people in practice population		
(N_countrie_*_s_* =34; N_GP_*_s_* =6,670)		
- below average	7.5%	ns
- average	9.1%	ns
- above average	11.9%	*p* = 0.04
Disadvantaged people in practice population		
(N_countrie_*_s_* =34; N_GP_*_s_* =6,807)		
- below average	6.5%	ns
- average	8.1%	ns
- above average	9.2%	ns
Practice type		
(N_countrie_*_s_* =33^a^; N_GP_*_s_* =6,968)		
- shared accommodation	6.9%	ns
- single-handed	8.8%	ns

^a^This information is lacking for Portugal.

### Relationships with patient experiences

We briefly present analyses of patient experiences, regarding communication, continuity, comprehensiveness, and accessibility (Supplementary Tables S1–S4). Experience of discrimination will be discussed in more detail.

Patient experiences of core dimensions of primary care were not associated with GPs’ migration status, except for accessibility in Model 1 (see Supplementary Table S2) where patients of immigrant GPs experienced lower accessibility. This relationship was non-significant in the next model, possibly because practices of immigrant GPs had above average ethnic minority people who experienced lower accessibility (see model 2 in Supplementary Table S2). First-generation migrant patients experienced worse communication and accessibility and second-generation migrant patients experienced worse accessibility and comprehensiveness of care (see model 3 in Tables S1–S4). No interaction terms modelling migration status concordance reached significance.

Overall, few patients reported experiencing discrimination from GP or practice staff (Table 2). ‘Don’t know’ responses were relatively high, especially as to whether or not they felt other patients were treated preferentially.

**Table 2.** Items measuring patient experiences with discrimination (overall percentages over all countries).

	Yes	No	Don’t know	Missing
	percentage (n)	percentage (n)	n	n
The doctor or staff acted negatively to you	3.5% (2,137)	96.5% (59,515)	1,092	908
Other patients were treated better than you	3.2% (1,567)	96.7% (45,981)	14,907	1,197
The doctor or staff showed disrespect because of your ethnic background	1.1% (641)	98.9% (59,158)	2,193	1,660

The reliability at GP level of the scale constructed from these items was 0.61 and at country level 0.55, which, although low, indicated that combining the three items provided information about GPs and countries. The intraclass correlation (ICC) showed that 56% of total variation was at GP level and a further 11% was at country level (bottom part of Table S5). At patient level, the reliability was very low: 0.17. The reason for this may be the relatively small number of nine patients per GP, further attenuated by ‘don’t know’ and missing responses.

There was no association between discrimination and whether or not GPs were born abroad (see Table 3). Experienced discrimination was related to other variables at GP, practice and patient level (see Table S5). Model 3 (full model without interaction terms) shows that patients of female GPs and in practices with higher than average ethnic minority people experienced more discrimination. Of the patient characteristics worse self-rated health and having one or more chronic conditions was related to more experienced discrimination. Higher educational level and middle or high income was associated with less experienced discrimination.

**Table 3.** Multilevel linear regression analysis of patient experiences with discrimination by GP or practice staff (N_countries_ =32^a^; N_practices_ =5,618; N_patients_ =45,525). Reduced table, for full Table see S5.

Variable	Model 1	Model 2^b^	Model 3^c^	Model 4
*Fixed part*	Coefficient (se)	Coefficient (se)	Coefficient (se)	Coefficient (se)
Constant	0.06 (0.01)	0.06 (0.009)	0.06 (0.02)	0.06 (0.02)
GP born in this country (1 = yes)	−0.0005 (0.006)	0.001 (0.006)	0.002 (0.006)	0.0008 (0.006)
Patients’ migration status (ref = non-migrant) - first generation migrant			0.009 (0.002)**	0.0005 (0.004)
- second generation migrant			0.004 (0.002)	0.006 (0.005)
*Interaction*				
GP born in this country and first generation migrant				0.01 (0.005)*
GP born in this country and second generation migrant				−0.003 (0.006)

*p < 0.05.

**p < .01.

^a^Data for Portugal are missing because of lacking information on practice type; data for Australia are missing because of lacking information on patients’ education.

^b^Model 2 additionally includes GPs’ age, sex, and practice location.

^c^Model 3 additionally includes patient characteristics.

First-generation migrants experienced more discrimination, but this was independent of their GP’s migration status (Model 3). Second-generation migrants did not experience more discrimination than ‘native’ patients. Model 4 shows no change in the main effects of GPs migrant status and of being second generation migrant, compared to model 3. For second-generation migrants, whether they saw a ‘native’ or immigrant GP did not affect their experience of discrimination. This was different for first-generation migrants. In model 4, the main effect disappears and moves to the interaction effect. Hence, first-generation migrants more often experienced discrimination when they were seen by a ‘native’ GP but less if they were seen by an immigrant GP.

The two sensitivity analyses (not reported in tables) do not show a stronger concordance effect and do otherwise not differ from the main analysis reported in Table S5. We used the model based on the single item ‘The doctor or staff acted negatively to you’ (which was used in one of the sensitivity analyses) to transform the interaction term into a percentage. The percentage experiencing that the doctor or staff acted negatively is 1.5 percentage points higher in the model with the interaction term, compared to the model without the interaction term (Model 3: sum of intercept + GP born in this country + patient first generation migrant is 2.5%, and Model 4: sum of intercept + GP born in this country + patient first generation migrant + interaction (GP born in this country and first generation migrant) is 4.04%).

## Discussion

### Summary

Regarding our first research question, proportions of immigrant GPs varied widely and they served populations higher in ethnic minority people.

Regarding the second research question, patient experiences in the core dimensions of primary care quality were not associated with whether GPs were native or born abroad. We found less positive experiences among patients with a migration background. Few patients experienced discrimination from their GP or staff, but first-generation migrants were more likely to, with variation between countries. Our analysis added the concordance in migration status of GPs and patients, and on the patient side divided into first- and second-generation migrants. We did not find concordance effects for the patient experiences in the core dimensions of primary care quality, but first-generation migrants more often experienced discrimination when they were seen by a ‘native’ GP but not when seen by an immigrant GP. However, although the interaction effect is significant, the percentage first-generation migrants who experienced discrimination when they were seen by a ‘native’ GP, is still small.

### Comparison with existing literature

Higher proportions people from ethnic minorities in foreign born GPs’ practices confirms previous findings [5]. We did not find high rates of patient responders who experienced discrimination from their GP or practice staff.

A previous analysis of the QUALICOPC data by Hanssens *et al.* also reported less positive experiences among patients with a migration background, but using partly different outcome indicators [11]. They also described differences between countries in experienced discrimination by patients. However, they did not analyse concordance of migration status of GPs and patients.

Concordance may be the result of the preferences of patients or of the availability of GPs with different backgrounds and patients who experienced discrimination in the past, may have switched to another GP or practice. The effects of concordance may differ according to whether patients were able to follow their preference and this may be a condition for positive effects [23]. Discordance may also be a choice for some patients under certain conditions [24].

The concordance effect with regard to experienced discrimination is more likely to be due to unconscious bias rather than explicit discrimination among GPs or staff. If the latter was the case, we would have also found this in second generation migrant responders. A possible explanation is a lack of cultural awareness skills among GPs and staff when relating to patients with a different cultural background or language [2]. In contrast there was no concordance effect in experience of doctor-patient communication, which could be expected if the explanation lies in skills for relating to patients with different backgrounds or language [25]. The concordance effect was found in a relatively small group of GPs and patients, both with a migration background. Our data from many GPs and patients made it possible to isolate this effect which may not be found with smaller samples.

Country characteristics might influence effects of migration status concordance. Countries with small migrant populations (including GPs) might show greater concordance effects, but because of lower numbers of both GPs and patients with a migration background, the power to detect a concordance effect is also reduced. This explains why we chose not to test three-way interactions, including country characteristics, although such characteristics may be related to concordance. Concordance effects might also be related to cultural influences. Keshnet, for example, found that in a collectivist minority culture (in this case in Israel) fear of confidentiality breaches by a physician from the same close-knit community may lead to preference for discordance [24].

The sample of countries contains a number of different primary care models and vary in the strength of primary care. However, we did not find clues in the literature to develop hypotheses about the potential impact of (primary) healthcare systems on the position of migrant GPs or on the effect of migration status concordance.

Immigrant GPs may also experience discrimination [4,26] and this may enhance awareness of discrimination and cultural sensitivity [16]. While immigrant GPs often fill gaps in care provision in underserved areas, their experiences of discrimination or differences in work culture may affect their job experience [26,27].

### Strengths and limitations

The analysis benefits from the large number of countries, GPs and patients included, linking data from GPs and patients enabling analysis of migration status concordance. Patient questionnaires were available in the main minority languages, as applicable in the country. One limitation is that GPs’ migration status was based on a single question. We did not ascertain whether ‘native’ GPs were second generation migrants or where GPs received their medical education.

There is a possibility of selection bias if immigrant GPs would be less inclined to respond. We had no data on the number of immigrant GPs in the GP population by country, but OECD Health Data had the percentage of foreign trained physicians by country for 25 countries included in QUALICOPC. There was a strong correlation (Spearman rank 0.83) between the percentage of immigrant GPs (from QUALICOPC) and the percentage of foreign trained physicians (from OECD). This does not rule out self-selection of immigrant GPs in QUALICOPC, but suggests that if present, it occurs to similar degrees in participating countries. Self-selection of patients was also possible, both in terms of participating in the study and in terms of selecting a GP that fitted their preferences.

The study populations of GPs were representative with regard to the distribution of age and sex of GPs in the respective countries [17]. The QUALICOPC study was conducted mainly in European countries and mostly among member states of the European Union; hence, mostly high-income countries. This limits the generalisability of the results. We do not know whether the findings, will also be valid in low- and middle-income countries and outside of Europe.

The measurement of patient experience of discrimination only addressed negative discrimination and one of the items explicitly addresses disrespect because of patients’ ethnic background; ethnic majority people may have found it difficult to answer this question. Few patients reported experiencing discrimination which may underestimate the real situation. Patients were asked to participate directly after the consultation by a fieldworker who was most probably a native of the country and might have been perceived as belonging to the practice. We do not know whether or not interpreters were involved in the consultations that were included in our study.

Our measurement of concordance was migration background concordance in a broad sense. We did not know the country of origin of both GP and patient (but if we did, the problem of the number of concordant pairs would be bigger). Also, we only explored concordance in one dimension instead of multiple dimensions [28].

A further limitation is that the data are by now somewhat old. Practice type and location of migrant GPs may have changed over time as well as the experience of discrimination of patients with a migrant background, as may public attitudes towards and discrimination against immigrants in general. The inflow of migrants in European countries has increased and their countries of origin have changed. Attitudes towards immigration may have changed also under the influence of increased inflow and (economic) uncertainties [29]. However, for the effect of migration status concordance, we think this is less of a limitation.

### Implications for research and practice

This study shows that patient experiences in key areas of primary care – accessibility, continuity, comprehensiveness and coordination – were not affected by migrant status of GPs. Nevertheless, health workforce mobility may affect health system performance and care quality in both country of origin and host country. Many countries expect GP shortages for years to come because of retirement and migration. Immigrant GPs are seen as a solution in some countries, whereas problems of medical workforce shortage are increased in countries with outward migration of doctors.

## Conclusion

Most research on practice location and practice patterns of immigrant GPs is based on single countries. This study uses data from 34 (mainly) European countries. Proportions of immigrant GPs varied widely and they served populations higher in ethnic minority people. Migration background of patients is associated with their self-reported experiences with the GP consultation. While experiences of discrimination by GPs or practice staff were uncommon, first-generation migrant patients experienced more discrimination. Primary care should provide safe access to non-discriminatory treatment. GPs should be aware of their own unconscious biases and this should be addressed during medical and GP training. Migration status (dis)concordance merits more research attention in purpose designed studies.

## Supplementary Material

Supplemental Material

## References

[1] Williams GA, Jacob G, Rakovac I, et al. Health professional mobility in the WHO European region and the WHO global code of practice: data from the joint OECD/EUROSTAT/WHO-Europe questionnaire. Eur J Public Health. 2020;30(Suppl_4):iv5–iv11. 32894282 10.1093/eurpub/ckaa124 PMC7526770

[2] Phung V-H, Asghar Z, Matiti M, et al. Understanding how Eastern european migrants use and experience UK health services: a systematic scoping review. BMC Health Serv Res. 2020;20(1):173. 32143703 10.1186/s12913-020-4987-z PMC7059702

[3] Lancee B. Ethnic discrimination in hiring: comparing groups across contexts. Results from a cross-national field experiment. J Ethn Migr Stud. 2019; 47:1–20. Published online: 24 Jun 2019.

[4] Klingler C, Marckmann G. Difficulties experienced by migrant physicians working in German hospitals: a qualitative interview study. Hum Resour Health. 2016;14(1):57. 27662831 10.1186/s12960-016-0153-4 PMC5034673

[5] Diaz E, Raza A, Sandvik H, et al. Immigrant and native regular general practitioners in Norway. A comparative registry based observational study. Eur J Gen Pract. 2014;20(2):93–99. 24000813 10.3109/13814788.2013.823600

[6] Goodfellow A, Ulloa JG, Dowling PT, et al. Predictors of primary care physician practice location in underserved urban or Rural Areas in the United States: a systematic literature review. Acad Med. 2016;91(9):1313–1321. 27119328 10.1097/ACM.0000000000001203 PMC5007145

[7] Wismar M, Maier CB, Glinos IA, Dussault G, Figueras J (eds). Health professional mobility and health systems: evidence from 17 European countries. Copenhagen: WHO, European Observatory on Health Systems and Policies; 2011.

[8] Verma P, Ford JA, Stuart A, et al. A systematic review of strategies to recruit and retain primary care doctors. BMC Health Serv Res. 2016;16:126. 27067255 10.1186/s12913-016-1370-1 PMC4828812

[9] Schäfer WLA, Boerma WGW, Schellevis FG, et al. GP practices as a one-stop shop: how do patients perceive the quality of care? A cross-sectional study in thirty-four countries Health Serv Res. 2018;53(4):2047–2063. 29285763 10.1111/1475-6773.12754 PMC6051984

[10] Hanssens LGM, Detollenaere J, Hardyns W, et al. Access, treatment and outcomes of care: a study of ethnic minorities in Europe. Int J Public Health. 2016;61(4):443–454. 27032868 10.1007/s00038-016-0810-3

[11] Hanssens LGM, Detollenaere J, Van Pottelberge A, et al. Perceived discrimination in primary healthcare in Europe: evidence from the cross-sectional QUALICOPC study. Health Soc Care Community. 2017;25(2):641–651. 27112973 10.1111/hsc.12353

[12] Eggermont D, Smit MAM, Kwestroo GA, et al. The influence of gender concordance between general practitioner and patient on antibiotic prescribing for sore throat symptoms: a retrospective study. BMC Fam Prac. 2018;19:175. 10.1186/s12875-018-0859-6 PMC6240216 30447685

[13] Shen MJ, Peterson EB, Costas-Muñiz R, et al. The effects of race and racial concordance on patient-physician communication: a systematic review of the literature. J Racial Ethn Health Disparities. 2018;5(1):117–140. 28275996 10.1007/s40615-017-0350-4 PMC5591056

[14] Strumpf EC. Racial/ethnic disparities in primary care: the role of physician-patient concordance. Med Care. 2011;49(5):496–503. 21430577 10.1097/MLR.0b013e31820fbee4

[15] Phillips KL, Chiriboga DA, Jang Y. Satisfaction with care: the role of patient-provider racial/ethnic concordance and interpersonal sensitivity. J Aging Health. 2012;24(7):1079–1090. 22869897 10.1177/0898264312453068

[16] Hjörleifsson S, Hammer E, Díaz E. General practitioners' strategies in consultations with immigrants in Norway-practice-based shared reflections among participants in focus groups. Fam Pract. 2018;35(2):216–221. 29029132 10.1093/fampra/cmx097

[17] Groenewegen PP, Greẞ S, Schäfer W. General Practitioners' Participation in a Large, Multicountry Combined General Practitioner-Patient Survey: Recruitment Procedures and Participation Rate. Int J Family Med. 2016;2016:4929432. 27047689 10.1155/2016/4929432 PMC4800081

[18] Schäfer WL, Boerma WG, Kringos DS, et al. QUALICOPC, a multi-country study evaluating quality, costs and equity in primary care. BMC Fam Pract. 2011;12:115. 22014310 10.1186/1471-2296-12-115 PMC3206822

[19] Schäfer WL, Boerma WG, Kringos DS, et al. Measures of quality, costs and equity in primary care instruments developed to analyse and compare primary care in 35 countries. Qual Prim Care. 2013;21(2):67–79. 23735688

[20] Commonwealth Fund. Survey on disparities in quality of health care. Commonwealth Fund, New York: 2011. https://www.commonwealthfund.org/publications/surveys/2011/nov/2011-commonwealth-fund-international-health-policy-survey. Accessed 25 October 2020.

[21] Leyland AH, Groenewegen PP. Multilevel modelling for public health and health services research: health in context. New York: SpringerOpen; 2020. 33347097

[22] Raudenbush S. The quantitative assessment of neighborhood social environments. In Kawachi I, Berkman L (eds). Neighborhoods and health. Oxford: Oxford University Press; 2004.

[23] Schnittker J, Liang K. The promise and limits of racial/ethnic concordance in physician-patient interaction. J Health Polit Policy Law. 2006;31(4):811–838. 16971546 10.1215/03616878-2006-004

[24] Keshet Y. Ethnic discordance: Why do some patients prefer to be treated by physicians from other ethnic groups? Soc Sc Med. 2019;235:112358. 31196576 10.1016/j.socscimed.2019.112358

[25] Hagiwara N, Elston Lafata J, Mezuk B, et al. Detecting implicit racial bias in provider communication behaviors to reduce disparities in healthcare: Challenges, solutions, and future directions for provider communication training. Patient Educ Couns. 2019;102(9):1738–1743. 31036330 10.1016/j.pec.2019.04.023 PMC7269129

[26] Eneroth M, Gustafsson Sendén M, Schenck Gustafsson K, et al. Threats or violence from patients was associated with turnover intention among foreign-born GPs - a comparison of four workplace factors associated with attitudes of wanting to quit one's job as a GP. Scand J Prim Health Care. 2017;35(2):208–213. 28587508 10.1080/02813432.2017.1333319 PMC5499322

[27] Kuusio H, Heponiemi T, Vänskä J, et al. Psychosocial stress factors and intention to leave job: differences between foreign-born and finnish-born general practitioners. Scand J Public Health. 2013;41(4):405–411. 23508947 10.1177/1403494813477248

[28] Thornton RLJ, Powe NR, Roter D, et al. Patient-physician social concordance, medical visit communication and patients' perceptions of health care quality. Patient Educ Couns. 2011;85(3):e201–e208. 21840150 10.1016/j.pec.2011.07.015 PMC3217162

[29] Davidov E, Semyonov M. Attitudes toward immigrants in European societies. Int J Comparat Sociol. 2017;58(5):359–366.

