# Supplementary material for: Migrant GPs and patients: a cross-sectional study of practice characteristics, patient experiences and migration concordance
Source: Scand J Prim Health Care. 2022 May 14;40(2):181–9. doi: 10.1080/02813432.2022.2069719 (PMC9397456; doi:10.1080/02813432.2022.2069719)
Supplement: Supplemental Material [file IPRI_A_2069719_SM9959.docx]

Supplementary tables

Table S1: Multilevel linear regression analysis of patient experiences in doctor-patient communication (N_countries_ =32; N_practices_ =5,618; N_patients_ =45,988).

| **Variable** | **Model 0** | **Model 1** | **Model 2** | **Model 3** | **Model 4** |
| --- | --- | --- | --- | --- | --- |
| ***Fixed part*** | Coefficient (se) | Coefficient (se) | Coefficient (se) | Coefficient (se) | Coefficient (se) |
| Constant | 0.96 (0.003) | 0.96 (0.003) | 0.96 (0.003) | 0.95 (0.005) | 0.95 (0.005) |
| *GP and practice variables* |  |  |  |  |  |
| Born in this country (1=yes) |  | 0.0009 (0.001) | 0.0003 (0.001) | 0.0002 (0.001) | 0.0004 (0.001) |
| Age GP |  |  | -7.4e-005 (4.6e-005) | -6.66e-005 (4.7e-005) | -6.6e-005 (4.7e-005) |
| Sex GP (1=female) |  |  | 0.002 (.0009)* | 0.002 (.0009)* | 0.002 (.0009)* |
| Proportion of elderly in the practice |  |  | -0.002 (0.0006)** | -0.002 (0.0006)** | -0.002 (0.0006)** |
| Proportion of ethnic minority people in the practice |  |  | -0.002 (0.0006)** | -0.002 (0.0006)** | -0.002 (0.0006)** |
| Proportion of disadvantaged people in the practice |  |  | -0.0007 (0.0006) | -0.0006 (0.0006) | -0.0005 (0.0006) |
| Practice type (single-handed=1) |  |  | -0.001 (0.001) | -0.001 (0.001) | -0.001 (0.001) |
| Urbanisation (big city=ref)  - suburbs |  |  | 0.001 (0.001) | 0.001 (0.001) | 0.001 (0.001) |
| - town |  |  | 0.0002 (0.001) | 0.0003 (0.001) | 0.0003 (0.001) |
| - mixed urban-rural |  |  | 0.001 (0.001) | 0.001 (0.001) | 0.001 (0.001) |
| - rural |  |  | 0.003 (0.001)* | 0.004 (0.001)** | 0.004 (0.001)** |
| *Patient variables* |  |  |  |  |  |
| Self-rated health (1-4) |  |  |  | -0.0005 (0.0003)* | -0.0005 (0.0003)* |
| Chronic disease (1=yes) |  |  |  | -1.4e-005 (0.0004) | -1.1e-005 (0.0004) |
| Patients’ sex (1=female) |  |  |  | 0.0005 (0.0003)* | 0.0005 (0.0003)* |
| Patients’ education (ref=low)  - middle |  |  |  | 0.001 (0.0005)** | 0.001 (0.0005)** |
| - high |  |  |  | 0.003 (0.0005)** | 0.003 (0.0005)** |
| Patients’ income (ref=low)  - middle |  |  |  | 0.0005 (0.0004) | 0.0005 (0.0004) |
| - high |  |  |  | 0.0009 (0.0007) | 0.0009 (0.0007) |
| Patients’ age |  |  |  | -4.4e-005 (1.2e-005)** | -4.4e-005 (1.2e-005)** |
| Patients’ migration status (ref=non-migrant)  - first generation migrant |  |  |  | -0.002 (0.0007)** | -0.002 (0.002) |
| - second generation migrant |  |  |  | -0.001 (0.0009) | 0.0002 (0.002) |
| *Interaction* |  |  |  |  |  |
| GP born in this country and first generation migrant |  |  |  |  | -0.0009 (0.002) |
| GP born in this country and second generation migrant |  |  |  |  | -0.002 (0.002) |
| ***Random part*** |  |  |  |  |  |
| ICC GPs (%) | 34.8 | 34.8 | 34.8 | 35.3 | 35.3 |
| ICC countries (%) | 12.2 | 12.3 | 11.9 | 10.7 | 10.7 |

*p < 0.05

**p < 0.01

^1^ Data for Portugal are missing because of lacking information on practice type; data for Australia are missing because of lacking information on patients’ education.

Table S2: Multilevel linear regression analysis of patient experiences in accessibility (N_countries_ =32; N_practices_ =5,619; N_patients_ =46,270).

| **Variable** | **Model 0** | **Model 1** | **Model 2** | **Model 3** | **Model 4** |
| --- | --- | --- | --- | --- | --- |
| ***Fixed part*** | Coefficient (se) | Coefficient (se) | Coefficient (se) | Coefficient (se) | Coefficient (se) |
| Constant | 0.85 (0.01)** | 0.85 (0.01)** | 0.85 (0.01)** | 0.84 (0.01)** | 0.84 (0.01)** |
| *GP and practice variables* |  |  |  |  |  |
| Born in this country (1=yes) |  | 0.008 (0.003)* | 0.006 (0.003) | 0.005 (0.003) | 0.006 (0.003) |
| Age GP |  |  | 6.9e-005 (0.0001) | 6.6e-005 (0.0001) | 6.6e-005 (0.0001) |
| Sex GP (1=female) |  |  | -0.002 (0.002) | -0.002 (0.002) | -0.002 (0.002) |
| Proportion of elderly in the practice |  |  | -0.001 (0.001) | -0.002 (0.001) | -0.002 (0.001) |
| Proportion of ethnic minority people in the practice |  |  | -0.006 (0.001)** | -0.006 (0.001)** | -0.006 (0.001)** |
| Proportion of disadvantaged people in the practice |  |  | -0.0009 (0.001) | -0.0007 (0.001) | -0.0007 (0.001) |
| Practice type (single-handed=1) |  |  | 0.002 (0.002) | 0.002 (0.002) | 0.002 (0.002) |
| Urbanisation (big city=ref)  - suburbs |  |  | 0.005 (0.003) | 0.005 (0.003) | 0.005 (0.003) |
| - town |  |  | 0.009 (0.003)** | 0.009 (0.003)** | 0.009 (0.003)** |
| - mixed urban-rural |  |  | 0.01 (0.003)** | 0.01 (0.003)** | 0.01 (0.003)** |
| - rural |  |  | 0.02 (0.003)** | 0.02 (0.003)** | 0.02 (0.003)** |
| *Patient variables* |  |  |  |  |  |
| Self-rated health (1-4) |  |  |  | -0.001 (0.0001)** | -0.001 (0.0001)** |
| Chronic disease (1=yes) |  |  |  | 0.0002 (0.0002) | 0.0002 (0.0002) |
| Patients’ sex (1=female) |  |  |  | 0.0008 (0.001) | 0.0008 (0.001) |
| Patients’ education (ref=low)  - middle |  |  |  | 0.0009 (0.0002)** | 0.0009 (0.0002)** |
| - high |  |  |  | 0.001 (0.0003)** | 0.001 (0.0003)** |
| Patients’ income (ref=low)  - middle |  |  |  | 0.002 (0.0002)** | 0.002 (0.0002)** |
| - high |  |  |  | 0.001 (0.0003)** | 0.001 (0.0003)** |
| Patients’ age |  |  |  | 6.5e-005 (6.3e-006)** | 6.5e-005 (6.3e-006)** |
| Patients’ migration status (ref=non-migrant)  - first generation migrant |  |  |  | -0.004 (0.0004)** | -0.003 (0.0008)** |
| - second generation migrant |  |  |  | -0.001 (0.0004)** | -0.0008 (0.001) |
| *Interaction* |  |  |  |  |  |
| GP born in this country and first generation migrant |  |  |  |  | -0.001 (0.0009) |
| GP born in this country and second generation migrant |  |  |  |  | -0.0005 (0.001) |
| ***Random part*** |  |  |  |  |  |
| ICC GPs (%) | 52.9 | 52.5 | 53.1 | 53.4 | 53.4 |
| ICC countries (%) | 43.3 | 43.6 | 42.9 | 42.6 | 42.6 |

*p < 0.05

**p < 0.01

^1^ Data for Portugal are missing because of lacking information on practice type; data for Australia are missing because of lacking information on patients’ education.

Table S3: Multilevel linear regression analysis of patient experiences in continuity of care (N_countries_ =32; N_practices_ =5,618; N_patients_ =46,135).

| **Variable** | **Model 0** | **Model 1** | **Model 2** | **Model 3** | **Model 4** |
| --- | --- | --- | --- | --- | --- |
| ***Fixed part*** | Coefficient (se) | Coefficient (se) | Coefficient (se) | Coefficient (se) | Coefficient (se) |
| Constant | 0.90 (0.02) | 0.90 (0.02) | 0.90 (0.02) | 0.87 (0.03) | 0.87 (0.03) |
| *GP and practice variables* |  |  |  |  |  |
| Born in this country (1=yes) |  | 0.006 (0.004) | 0.006 (0.004) | 0.004 (0.004) | 0.005 (0.004) |
| Age GP |  |  | 0.0007 (0.0001)** | 0.0006 (0.0001)** | 0.0006 (0.0001)** |
| Sex GP (1=female) |  |  | 0.004 (0.003) | 0.004 (0.003) | 0.004 (0.003) |
| Proportion of elderly in the practice |  |  | -0.003 (0.002) | -0.006 (0.002)** | -0.006 (0.002)** |
| Proportion of ethnic minority people in the practice |  |  | -0.002 (0.002) | -0.001 (0.002) | -0.001 (0.002) |
| Proportion of disadvantaged people in the practice |  |  | 0.0009 (0.002) | 0.0009 (0.002) | 0.0009 (0.002) |
| Practice type (single-handed=1) |  |  | 0.006 (0.003) | 0.006 (0.003) | 0.006 (0.003) |
| Urbanisation (big city=ref)  - suburbs |  |  | 0.01 (0.004)* | 0.01 (0.004)* | 0.01 (0.004)* |
| - town |  |  | 0.02 (0.004)** | 0.02 (0.004)** | 0.02 (0.004)** |
| - mixed urban-rural |  |  | 0.02 (0.004)** | 0.02 (0.004)** | 0.02 (0.004)** |
| - rural |  |  | 0.03 (0.004)** | 0.03 (0.004)** | 0.03 (0.004)** |
| *Patient variables* |  |  |  |  |  |
| Self-rated health (1-4) |  |  |  | 0.003 (0.0007)** | 0.003 (0.0007)** |
| Chronic disease (1=yes) |  |  |  | 0.01 (0.001)** | 0.01 (0.001)** |
| Patients’ sex (1=female) |  |  |  | 0.002 (0.002) | 0.002 (0.002) |
| Patients’ education (ref=low)  - middle |  |  |  | -0.0006 (0.001) | -0.0006 (0.001) |
| - high |  |  |  | -0.002 (0.002) | -0.002 (0.002) |
| Patients’ income (ref=low)  - middle |  |  |  | 0.003 (0.001)** | 0.003 (0.001)** |
| - high |  |  |  | 0.006 (0.002)** | 0.006 (0.002)** |
| Patients’ age |  |  |  | 0.0006 (3.6e-005)** | 0.0006 (3.6e-005)** |
| Patients’ migration status (ref=non-migrant)  - first generation migrant |  |  |  | -0.005 (0.002)* | -0.006 (0.005) |
| - second generation migrant |  |  |  | -0.003 (0.003) | 0.003 (0.006) |
| *Interaction* |  |  |  |  |  |
| GP born in this country and first generation migrant |  |  |  |  | 0.001 (0.005) |
| GP born in this country and second generation migrant |  |  |  |  | -0.007 (0.007) |
| ***Random part*** |  |  |  |  |  |
| ICC GPs (%) | 25.2 | 25.1 | 25.2 | 25.4 | 25.4 |
| ICC countries (%) | 37.3 | 37.4 | 36.5 | 35.8 | 35.8 |

*p < 0.05

**p < 0.01

^1^ Data for Portugal are missing because of lacking information on practice type; data for Australia are missing because of lacking information on patients’ education.

Table S4: Multilevel linear regression analysis of patient experiences in comprehensiveness of care (N_countries_ =32; N_practices_ =5,618; N_patients_ =46,000).

| **Variable** | **Model 0** | **Model 1** | **Model 2** | **Model 3** | **Model 4** |
| --- | --- | --- | --- | --- | --- |
| ***Fixed part*** | Coefficient (se) | Coefficient (se) | Coefficient (se) | Coefficient (se) | Coefficient (se) |
| Constant | 0.68 (0.02) | 0.68 (0.02) | 0.68 (0.02) | 0.65 (0.03) | 0.65 (0.03) |
| *GP and practice variables* |  |  |  |  |  |
| Born in this country (1=yes) |  | 0.005 (0.005) | 0.004 (0.005) | 0.004 (0.005) | 0.004 (0.005) |
| Age GP |  |  | 0.0002 (0.0002) | 0.0002 (0.0002) | 0.0002 (0.0002) |
| Sex GP (1=female) |  |  | 0.01 (0.003)** | 0.01 (0.003)** | 0.01 (0.003)** |
| Proportion of elderly in the practice |  |  | -0.004 (0.002) | -0.004 (0.002) | -0.004 (0.002) |
| Proportion of ethnic minority people in the practice |  |  | -0.002 (0.002) | -0.002 (0.002) | -0.002 (0.002) |
| Proportion of disadvantaged people in the practice |  |  | 0.004 (0.002) | 0.004 (0.002) | 0.004 (0.002) |
| Practice type (single-handed=1) |  |  | 0.001 (0.004) | 0.001 (0.004) | 0.001 (0.004) |
| Urbanisation (big city=ref)  - suburbs |  |  | 0.004 (0.005) | 0.004 (0.005) | 0.004 (0.005) |
| - town |  |  | 0.006 (0.004) | 0.006 (0.004) | 0.006 (0.004) |
| - mixed urban-rural |  |  | 0.01 (0.005)** | 0.01 (0.005)** | 0.01 (0.005)** |
| - rural |  |  | 0.02 (0.005)** | 0.02 (0.005)** | 0.02 (0.005)** |
| *Patient variables* |  |  |  |  |  |
| Self-rated health (1-4) |  |  |  | 0.0005 (0.0002)** | 0.0005 (0.0002)** |
| Chronic disease (1=yes) |  |  |  | 0.002 (0.0003)** | 0.002 (0.0003)** |
| Patients’ sex (1=female) |  |  |  | 0.002 (0.002) | 0.002 (0.002) |
| Patients’ education (ref=low)  - middle |  |  |  | -0.0000 (0.0003) | -0.0000 (0.0003) |
| - high |  |  |  | -0.0006 (0.0004) | -0.0006 (0.0004) |
| Patients’ income (ref=low)  - middle |  |  |  | -0.0002 (0.0003) | -0.0001 (0.0003) |
| - high |  |  |  | -0.0002 (0.0005) | -0.0002 (0.0005) |
| Patients’ age |  |  |  | 4.6e-005 (8.6e-006)** | 4.7e-005 (8.6e-006)** |
| Patients’ migration status (ref=non-migrant)  - first generation migrant |  |  |  | -0.0000 (0.0005) | -0.001 (0.001) |
| - second generation migrant |  |  |  | -0.001 (0.0006)* | -0.001 (0.001) |
| *Interaction* |  |  |  |  |  |
| GP born in this country and first generation migrant |  |  |  |  | 0.002 (0.001) |
| GP born in this country and second generation migrant |  |  |  |  | 0.0000 (0.002) |
| ***Random part*** |  |  |  |  |  |
| ICC GPs (%) | 56.0 | 55.9 | 56.5 | 57.6 | 57.6 |
| ICC countries (%) | 41.1 | 41.3 | 40.7 | 39.5 | 39.5 |

*p < 0.05

**p < 0.01

^1^ Data for Portugal are missing because of lacking information on practice type; data for Australia are missing because of lacking information on patients’ education.

Table S5: Multilevel linear regression analysis of patient experiences with discrimination by GP or practice staff (N_countries_ =32^1^; N_practices_ =5,618; N_patients_ =45,525).

| **Variable** | **Model 0** | **Model 1** | **Model 2** | **Model 3** | **Model 4** |
| --- | --- | --- | --- | --- | --- |
| ***Fixed part*** | Coefficient (se) | Coefficient (se) | Coefficient (se) | Coefficient (se) | Coefficient (se) |
| Constant | 0.06 (0.01) | 0.06 (0.01) | 0.06 (0.009) | 0.06 (0.02) | 0.06 (0.02) |
| *GP and practice variables* |  |  |  |  |  |
| Born in this country (1=yes) |  | -0.0005 (0.006) | 0.001 (0.006) | 0.002 (0.006) | 0.0008 (0.006) |
| Age GP |  |  | -0.0001 (0.0002) | -0.0001 (0.0002) | -0.0001 (0.0002) |
| Sex GP (1=female) |  |  | 0.009 (0.004)* | 0.009 (0.004)* | 0.009 (0.004)* |
| Share of elderly in the practice |  |  | 0.004 (0.002) | 0.005 (0.002) | 0.005 (0.002) |
| Share of ethnic minority people in the practice |  |  | 0.007 (0.003)* | 0.006 (0.003)* | 0.006 (0.003)* |
| Share of disadvantaged people in the practice |  |  | 0.005 (0.003)* | 0.004 (0.003) | 0.004 (0.003) |
| Practice type (single-handed=1) |  |  | 0.002 (0.004) | 0.002 (0.004) | 0.002 (0.004) |
| Urbanisation (big city=ref)  - suburbs |  |  | -0.001 (0.006) | -0.001 (0.006) | -0.001 (0.006) |
| - town |  |  | 0.005 (0.005) | 0.005 (0.005) | 0.005 (0.005) |
| - mixed urban-rural |  |  | 0.006 (0.005) | 0.006 (0.005) | 0.006 (0.005) |
| - rural |  |  | -0.01 (0.005) | -0.01 (0.005) | -0.01 (0.005) |
| *Patient variables* |  |  |  |  |  |
| Self-rated health (1-4) |  |  |  | 0.003 (0.0007)** | 0.003 (0.0007)** |
| Chronic disease (1=yes) |  |  |  | 0.003 (0.001)* | 0.003 (0.001)* |
| Patients’ sex (1=female) |  |  |  | 7.3e005 (0.0009) | 7.4e005 (0.0009) |
| Patients’ education (ref=low)  - middle |  |  |  | -0.001 (0.001) | -0.001 (0.001) |
| - high |  |  |  | -0.004 (0.001)** | -0.004 (0.001)** |
| Patients’ income (ref=low)  - middle |  |  |  | -0.007 (0.001)** | -0.007 (0.001)** |
| - high |  |  |  | -0.004 (0.002)* | -0.004 (0.002)* |
| Patients’ age |  |  |  | -0.0002 (3.2e-005)** | -0.0002 (3.2e-005)** |
| Patients’ migration status (ref=non-migrant)  - first generation migrant |  |  |  | 0.009 (0.002)** | 0.0005 (0.004) |
| - second generation migrant |  |  |  | 0.004 (0.002) | 0.006 (0.005) |
| *Interaction* |  |  |  |  |  |
| GP born in this country and first generation migrant |  |  |  |  | 0.01 (0.005)* |
| GP born in this country and second generation migrant |  |  |  |  | -0.003 (0.006) |
| ***Random part*** |  |  |  |  |  |
| ICC GPs (%) | 56.3 | 56.3 | 56.3 | 56.3 | 56.3 |
| ICC countries (%) | 10.8 | 10.8 | 10.5 | 10.5 | 10.5 |

*p < 0.05

**p < 0.01

^1^ Data for Portugal are missing because of lacking information on practice type; data for Australia are missing because of lacking information on patients’ education.
